# Soil nutritional status and biogeography influence rhizosphere microbial communities associated with the invasive tree *Acacia dealbata*

**DOI:** 10.1038/s41598-017-07018-w

**Published:** 2017-07-26

**Authors:** Casper N. Kamutando, Surendra Vikram, Gilbert Kamgan-Nkuekam, Thulani P. Makhalanyane, Michelle Greve, Johannes J. Le Roux, David M. Richardson, Don Cowan, Angel Valverde

**Affiliations:** 1 Centre for Microbial Ecology and Genomics, Department of Genetics, University of Pretoria, Pretoria, South Africa; 2 Department of Plant and Soil Sciences, University of Pretoria, Pretoria, South Africa; 3 Centre for Invasion Biology, Department of Botany & Zoology, Stellenbosch University, Stellenbosch, South Africa

## Abstract

Invasiveness and the impacts of introduced plants are known to be mediated by plant-microbe interactions. Yet, the microbial communities associated with invasive plants are generally poorly understood. Here we report on the first comprehensive investigation of the bacterial and fungal communities inhabiting the rhizosphere and the surrounding bulk soil of a widespread invasive tree, *Acacia dealbata*. Amplicon sequencing data indicated that rhizospheric microbial communities differed significantly in structure and composition from those of the bulk soil. Two bacterial (*Alphaproteobacteria* and *Gammaproteobacteria*) and two fungal (*Pezizomycetes* and *Agaricomycetes*) classes were enriched in the rhizosphere compared with bulk soils. Changes in nutritional status, possibly induced by *A*. *dealbata*, primarily shaped rhizosphere soil communities. Despite a high degree of geographic variability in the diversity and composition of microbial communities, invasive *A*. *dealbata* populations shared a core of bacterial and fungal taxa, some of which are known to be involved in N and P cycling, while others are regarded as plant pathogens. Shotgun metagenomic analysis also showed that several functional genes related to plant growth promotion were overrepresented in the rhizospheres of *A*. *dealbata*. Overall, results suggest that rhizosphere microbes may contribute to the widespread success of this invader in novel environments.

## Introduction

Invasive trees are a global concern because they pose a direct threat to natural ecosystems and cause important economic losses, although they also provide goods and services that sustain human populations^1^. Australian acacias are among the most invasive trees worldwide^2^. In their invasive ranges, acacias have had numerous and severe environmental impacts^3, 4^. For example, *Acacia dealbata* (silver wattle) is an aggressive invader in southern Europe, the Americas and southern Africa^2^. In South Africa, this species reduces water availability^5^, and alters faunal^6^ and microbial^7^ community diversity and composition. *Acacia dealbata* also reduces the abundance and cover of native plant species under its understory^8^ and modifies soil basal respiration and soil enzymatic activities^9^, two important proxies for ecosystem functioning.

Plant species traits (e.g., high seed outputs, adaptability), abiotic factors (e.g., similar climate and soil chemistry in native and invaded ranges), native range biogeography, and deliberate and accidental human intervention^10^, have been commonly used to explain why many acacia species have become invasive in new environments^11^.

It is also likely that rhizosphere microbes (native or introduced), such as bacteria and fungi, play an important role in the establishment and invasion success of acacias^12^. Microbial communities influence many important ecosystem processes, including plant nutrient acquisition and nitrogen and carbon cycling^13^, which regulate plant diversity and productivity. For example, acacias develop symbiotic associations with rhizobia that convert atmospheric nitrogen into plant available ammonium^14^. Recent work has shown that invasive acacias in South Africa’s Cape Floristic Region (CFR) associate with a diverse assemblage of *Bradyrhizobium* strains that are not commonly associated with native legumes^15^. Acacias also interact with arbuscular mycorrhizal (AMF) and/or ectomycorrhizal (EMF) fungi^16^ which modify the root system and enhance mobilization and the uptake of several essential elements, especially phosphorus^17^. On the other hand, microbial parasites and pathogens, through their effects on plant health and productivity, may impact negatively on plant invasiveness^18^.

Apart from the interactions of acacias with rhizobia and mycorrhiza, there is currently little knowledge about the identity of the overall soil microbial communities associated with acacia species. For instance, for *A*. *dealbata* only limited data are available from studies employing low resolution methods and/or low sample sizes^19, 20^ (but see ref. 7). In addition, most of these studies were carried out in soils in the proximity of plants, but not in the narrow zone of soil that surrounds and is most influenced by plant roots (i.e., the rhizosphere). As rhizosphere microbes can directly and/or indirectly affect the composition and biomass of plant communities^21^, increasing our knowledge on the root microbiome is important to better understand the factors contributing to plant invasiveness.

Here, we use Illumina MiSeq data (targeting 16S rRNA genes and ITS regions) and shotgun metagenomics to investigate microbial (bacterial and fungal) communities and functional processes associated with the roots of this species in various invasive populations of *A*. *dealbata* across South African grasslands, and compare them with those from the bulk soil. More specifically, we evaluate (i) how abiotic (i.e., soil chemistry) factors may shape the diversity and structure of these communities, (ii) identify prominent taxa that may contribute to the success of this invader and (iii) investigate whether rhizosphere and bulk soil microbial communities differ in their metabolic capacity.

## Results and Discussion

Analysing the microbial communities associated with the rhizospheres of *A*. *dealbata*, we obtained 2,444,152 and 694,434 total high-quality reads, which resulted in 29,678 and 14,471 OTUs (97% cut-off) for bacteria and fungi, respectively. The majority of all prokaryotic OTUs in the rhizosphere were also present in the bulk soils (Supplementary Fig. S2a), whereas a larger proportion of the eukaryotic OTUs were unique to the sample type (Supplementary Fig. S2b). For both bacterial and fungal communities the OTUs shared between rhizosphere and bulk soil samples accounted for the majority of the reads (97.2% and 93% respectively; Supplementary Fig. S2a,b). The percentage of OTUs shared between all eight sampling sites was considerably smaller for fungal data sets than for the bacterial data sets (1.6% and 12%, respectively). This is in agreement with the view that fungal communities generally show more geographic structure than bacterial communities^22^.

OTU accumulation curves indicated reasonable sequence saturation at a regional level, especially for bacteria (Supplementary Fig. S3). The levels of microbial diversity (richness, Shannon, inverse Simpson, Pielou’s evenness) tended to be higher in the bulk soil than in the rhizosphere samples, although they did not differ significantly (Supplementary Fig. S4). This is in contrast to previous studies^19^, which showed *A*. *dealbata-*invaded zones containing higher bacterial richness and lower fungal richness compared to uninvaded zones. However, it is now accepted that the community fingerprinting method used in that study (i.e., DGGE) is not well suited to accurately estimate microbial richness^23^.

**Figure 1:**
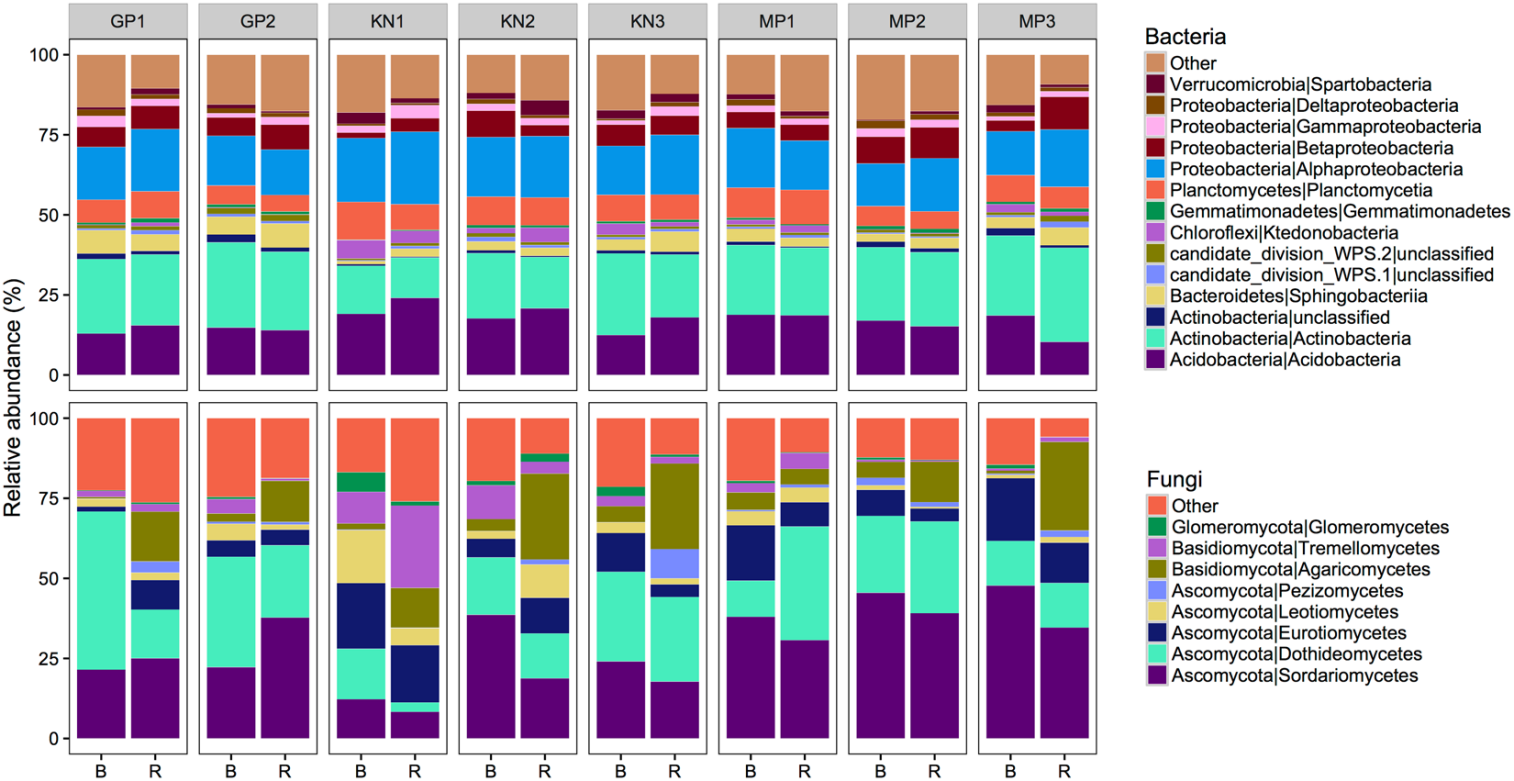
Mean relative abundances of taxa (phylum/class levels) within each location. The abundance of each taxon was calculated as the percentage of sequences per location for a given microbial group. The group ‘Other’ encompasses unclassified sequences together with classes representing ≤0.5% of total sequences. B, bulk soil; R, rhizosphere soil.

A total of 30 distinct bacterial phyla were detected across all samples. The most abundant sequences were affiliated with the phylum *Proteobacteria* (27% of total relative abundance), followed by *Actinobacteria* (23%), *Acidobacteria* (18%), *Planctomycetes* (8%) and *Bacteroidetes* (5%) (Fig. 1). These phyla have been shown to be widely represented in bulk and rhizosphere soil samples (e.g., see review by ref. 21), including those from other invasive species such as Japanese barberry^24^. At the class level, *Actinobacteria* (22%), *Acidobacteria* (17%), *Alphaproteobacteria* (17%), *Planctomycetia* (8%) and *Betaproteobacteria* (6%) were represented by the majority of sequences. Rhizosphere soil samples were enriched for *Alphaproteobacteria* (Kruskal-Wallis χ^2^ = 4.4; P < 0.05) and *Gammaproteobacteria* (Kruskal-Wallis χ^2^ = 7.4; P < 0.01), and depauperate in *Spartobacteria* (Verrucomicrobia; Kruskal-Wallis χ^2^ = 9.1; P < 0.01), compared with the bulk soil. Members of the *Alphaproteobacteria* and *Gammaproteobacteria* are usually defined as copiotrophic^25^; that is, they compete successfully when organic resources are abundant. The high abundance of these OTUs in the rhizosphere of *A*. *dealbata* plants could reflect their ability to proliferate in the presence of plant-derived polysaccharides. *Spartobacteria* are highly abundant in grassland soils^26^, probably because they associate with nematodes, which are typically abundant in grasslands^27^. Whether the decrease in *Spartobacteria* in this study results directly from the effects of wattles or indirectly through trophic cascades needs to be further investigated.

Fungal communities were dominated by the phylum *Ascomycota* (74%), while *Basidiomycota* represented only 16% of the reads. This corroborates what has been found across grasslands worldwide^28^. At the class level, the majority of the sequences could be assigned to *Sordariomycetes* (29%), *Dothideomycetes* (22%), *Agaricomycetes* (10%) and *Eurotiomycetes* (10%). Other groups such as *Pezizomycetes* (1.5%) and *Glomeromycetes* (AMF, 1.2%) were also represented, albeit in lower proportions. Rhizosphere soil samples were enriched for *Pezizomycetes* (Kruskal-Wallis χ^2^ = 11.0; P < 0.001) and *Agaricomycetes* (Kruskal-Wallis χ^2^ = 25.0; P < 0.001). High *Pezizomycetes* abundance has also been found in the rhizosphere of other trees such as willows^29^. The majority of *Agaricomycetes* sequences were identified as *Inocybe* sp., a widely distributed ectomycorrhizal fungus (EMF)^30^. This is not surprising, as *Inocybe* species have been found in the rhizosphere of other acacias^31^. *Glomeromycetes*, mainly the genera *Gigaspora*, *Acaulospora*, *Dentiscutata* and *Glomus*, were more abundant in bulk than in rhizosphere soils (Kruskal-Wallis χ^2^ = 6.4; P < 0.05). There are at least two possible explanations for this. First, it could be argued that AMF were less abundant in rhizosphere soils for historical reasons; that is, they were historically less dominant in the areas invaded by *A*. *dealbata*. However, we find this to be unlikely because historical differences in AMF abundance would be unlikely to be maintained over time at small spatial distances (10–20 m separated rhizosphere and bulk soil samples in a given location) due to the efficient dispersal of these fungi^32^. Therefore, it is likely that the lower AMF abundance results as a consequence of the invasion process^12^. The inhibition of some AMF by allelopathic compounds has previously been shown in other invasive plants^33^, but it is uncertain in *A*. *dealbata* where the allelopathic effects seem to be dependent on ecosystem type^34^. Alternatively, it has also been suggested that AMF in plants with dual associations (i.e., plants in association with both AMF and EMF) are only found in substantial abundance when these plants are growing in disturbed habitats or flooded soils, or are present as young seedlings^35^.

Soil chemistry differed significantly between rhizosphere and bulk soil samples (PERMANOVA F_1,76_ = 10.93; P < 0.001). Comparisons between rhizosphere and bulk soils showed that Total C, Total N, NH_4_ and NO_3_ values were higher for rhizosphere than for bulk soils (Supplementary Fig. S5). *Acacia dealbata* is able to alter the spatial distribution of nutrients in a wide variety of habitats^19, 36^ and our results are consistent with these observations. It has been suggested that the massive detritus production under the *A*. *dealbata* canopy results in increased accumulation of these nutrients in invaded soils^37^. Nitrogen levels are further improved due to the association of *A*. *dealbata* with N_2_-fixing bacteria. In contrast to what was shown in previous studies, we did not find *A*. *dealbata* to have lowered the pH and/or increased the P content of the rhizosphere soil. Since soil acidification and P increase seem to reach their maxima 10–25 years after invasion^20^, it is possible that our data reflect relatively recent invasion events.

**Figure 2:**
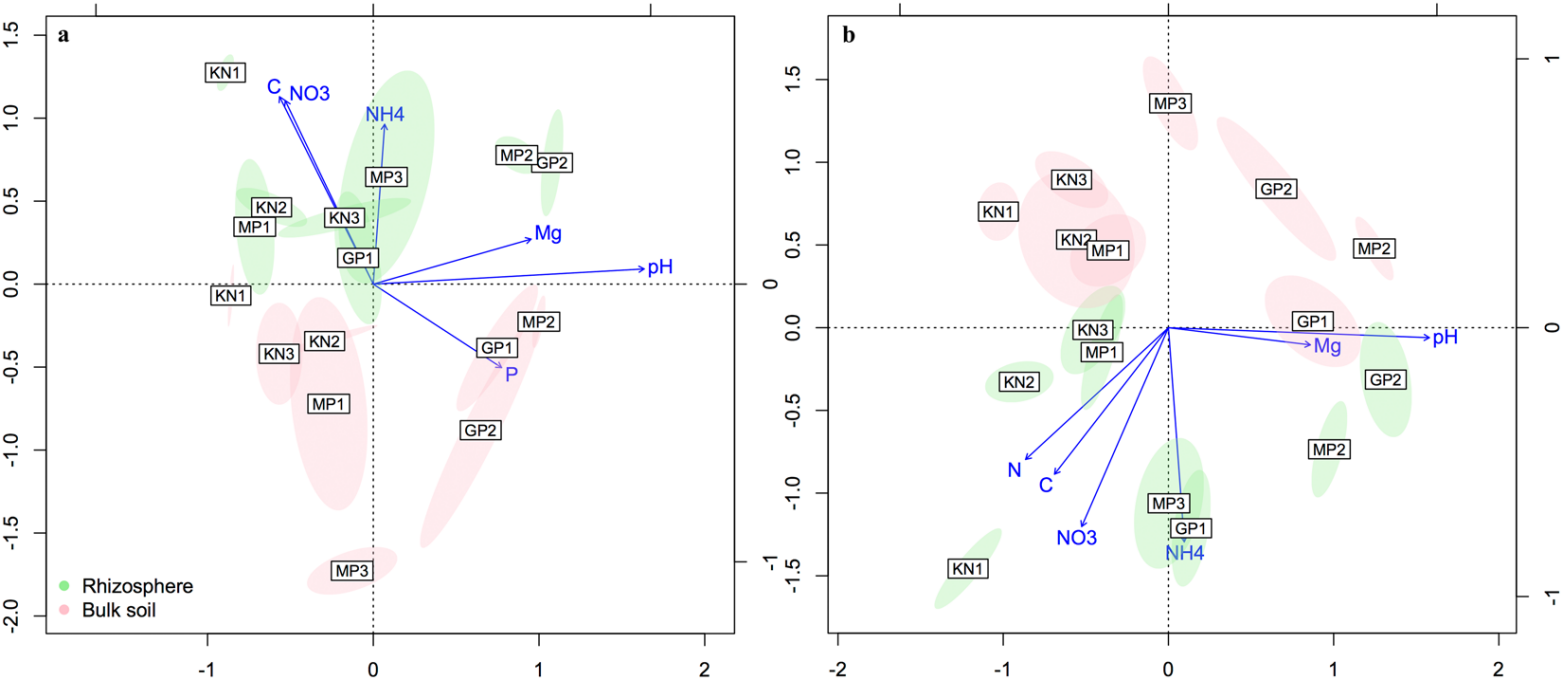
Distance-based redundancy analysis (db-RDA) biplot of (**a**) bacterial and (**b**) fungal communities and soil chemistry parameters. Only the environmental variables that significantly (P < 0.05) explained variability in microbial community structure are shown (arrows). The direction of the arrows indicates the direction of maximum change of that variable, whereas the length of the arrow is proportional to the rate of change. Instead of plotting each sampling point, ellipses are shown that represent the standard error around location centroids.

**Figure 3:**
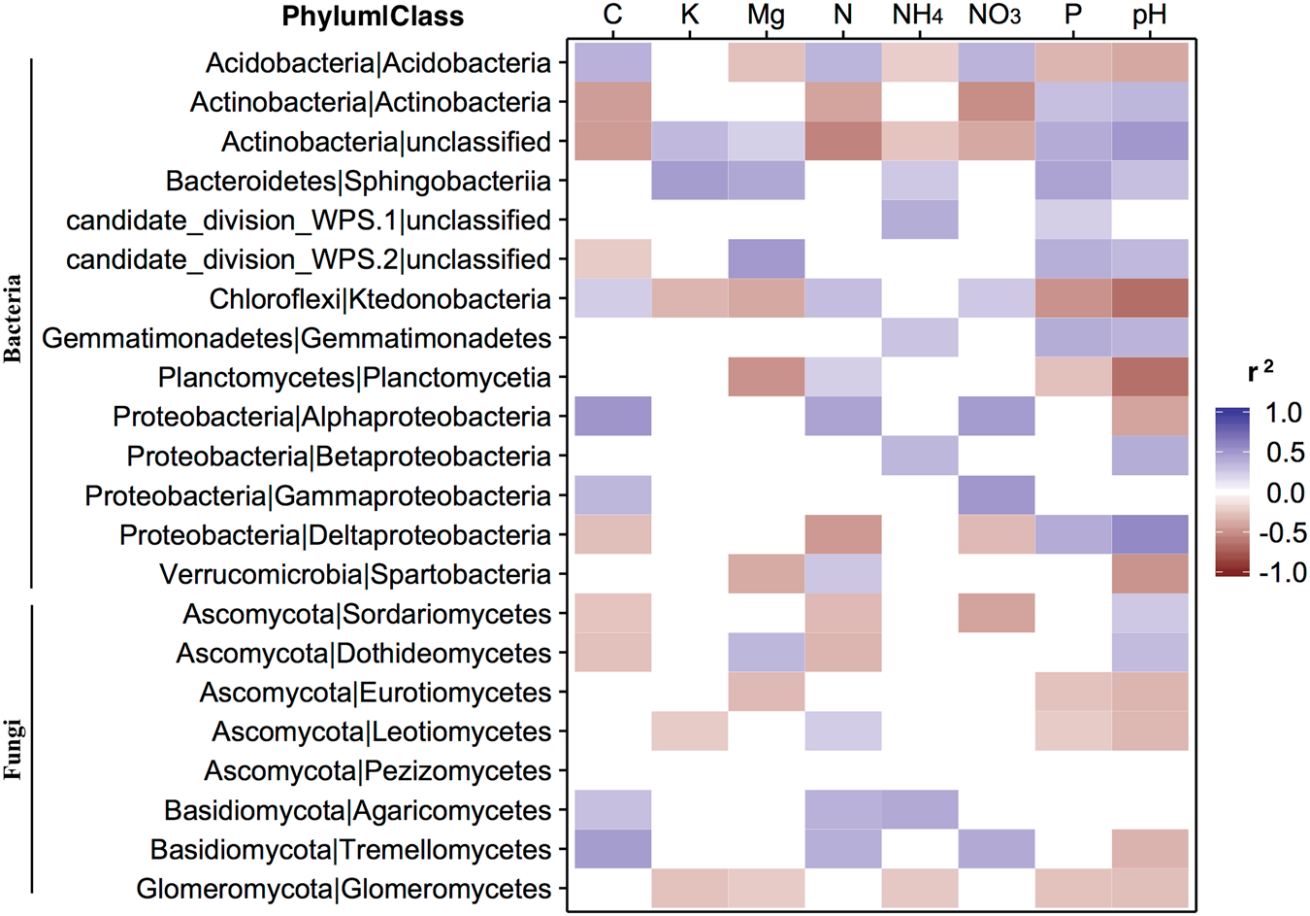
Correlation between soil properties (aggregate data for rhizosphere and bulk soils) and the different bacterial and fungal classes shown in Fig. 1. Significance level P < 0.05.

The presence of *A*. *dealbata* had a significant effect on microbial community structure and composition based on Bray-Curtis distances (PERMANOVA_Bacteria_ F_1,76_ = 3.44; PERMANOVA_Fungi_ F_1,76_ = 2.60; both P < 0.001). Similar patterns were observed for the 16S rRNA gene data set using unweighted (PERMANOVA_Bacteria_ F_1,76_ = 2.84; P < 0.001) and weighted (PERMANOVA_Bacteria_ F_1,76_ = 3.33; P < 0.001) UniFrac distances. We did not perform PERMANOVA for the ITS data set with UniFrac distances because of known issues with the accuracy of phylogenetic trees generated from this hypervariable region^38^. Redundancy analysis suggested that soil chemistry changes (especially in pH, C, NO_3_, NH_4_, P and Mg) around acacia roots played an important role in shaping the structure and composition of microbial communities (Fig. 2). Results showed that the different classes of bacteria and fungi were significantly correlated with these and other soil parameters (Fig. 3). Factors such as pH and nutrient status are the main drivers controlling composition and diversity of soil microbial communities^39^. Additional factors, for instance, the modification of soil structure by the root system^40^ and the release of allelopathic compounds^40, 41^ might also lead to changes in microbial communities.

**Figure 4:**
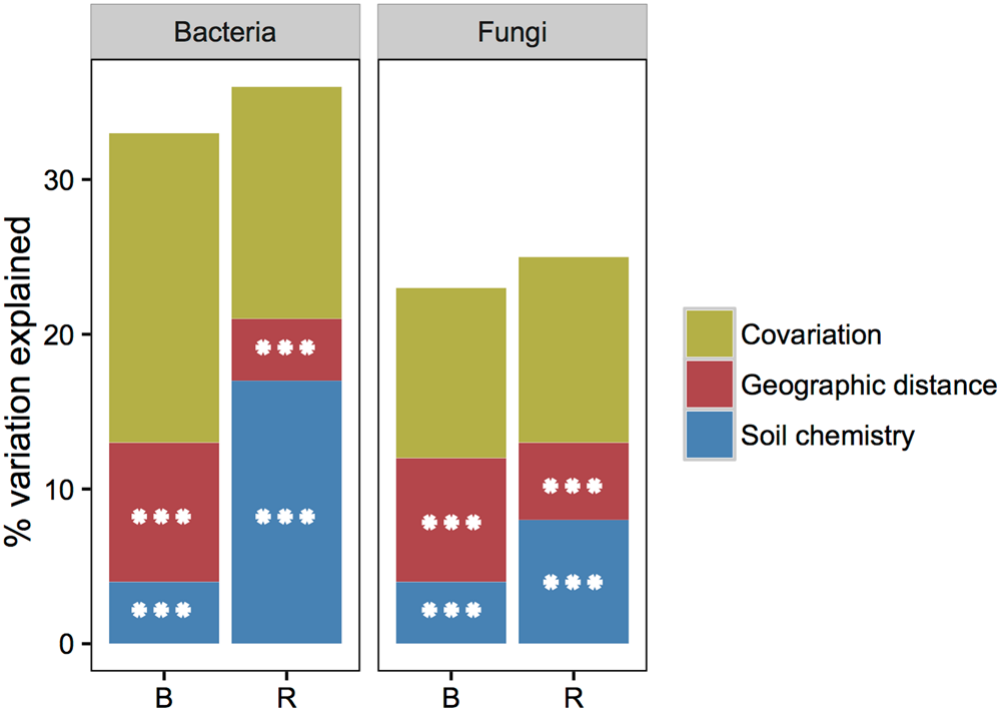
Partitioning of the variation in bacterial and fungal community structure. The specific effects of soil chemistry and geographic distance and the total co-variation (i.e., the variance jointly explained by the soil chemistry and geographic distance) are represented. Statistical significance is indicated by ***P < 0.001. B, bulk soil; R, rhizosphere soil.

The effects of soil chemistry on variation in microbial community structure were further investigated by taking into account the confounding effects of geography (spatial distance between samples). Variation partitioning models explained between 23–25% (fungi) and 33–36% (bacteria) of the community variation, with soil chemistry and spatial distance significantly explaining 4–17% and 4–9% of the variation, respectively (Fig. 4; P < 0.001, based on 999 Monte Carlo permutation tests). We note that a large proportion of the variation remains unexplained. Although environmental variables that were not recorded may contribute to the high fraction of unexplained variation, we speculate that this could also be caused by ecological drift (i.e., changes in species abundances caused by the stochastic processes). Ecological drift seems to be an important driver for microbial community assembly^42^. Interestingly, bulk soil communities were more affected by geographic distance than rhizosphere soil communities, contradicting previous findings^43^. Overall, these results suggest that spatial distance, soil properties and *A*. *dealbata*-mediated soil interactions shape the organization of microbial communities on a regional scale.

**Figure 5:**
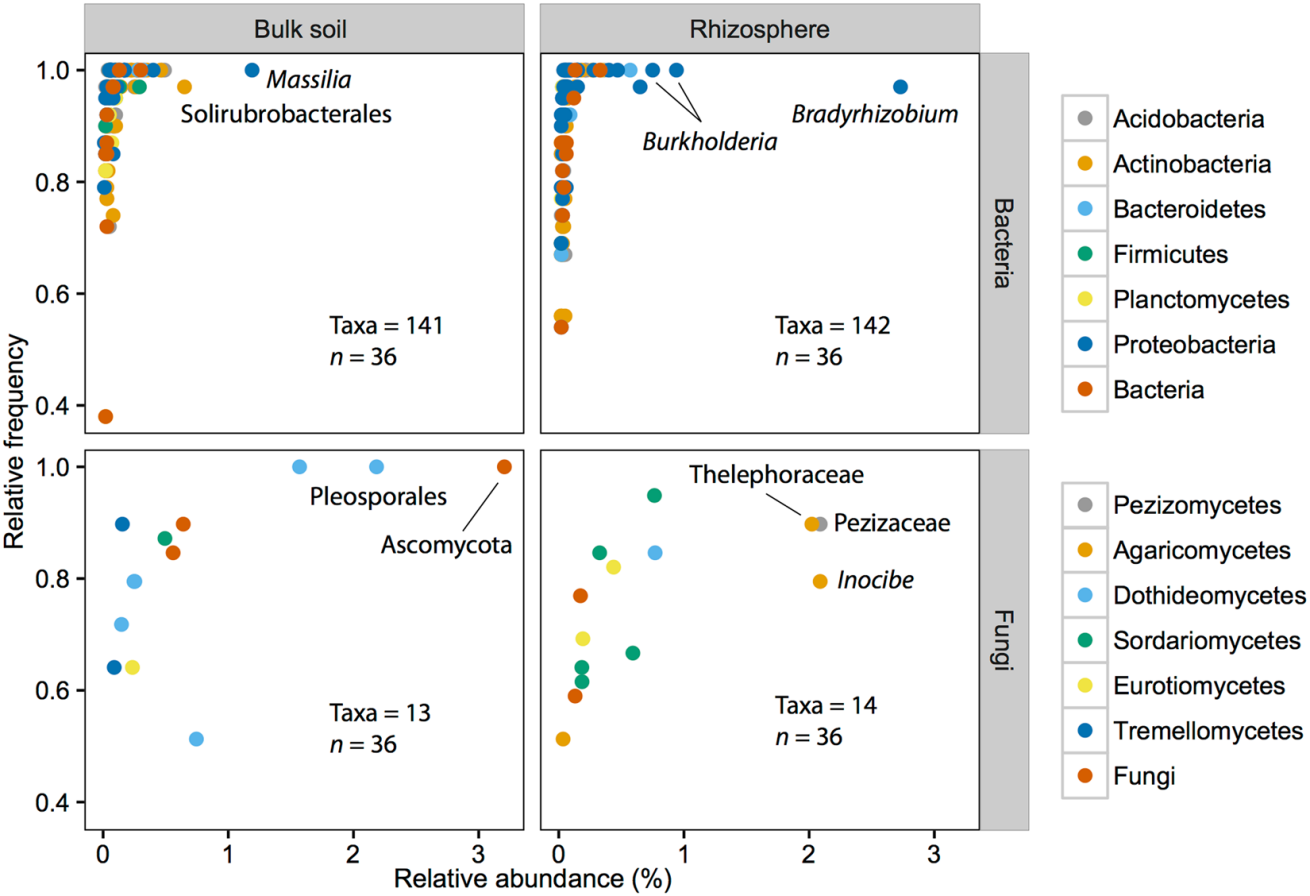
Relative frequency versus relative abundance of biomarker taxa, coloured according to phylum, for bulk and rhizosphere soils (logarithmic LDA score ≥2, P < 0.05). The number of biomarker taxa (OTUs) and the number of samples (*n*) are indicated in each plot.

Despite the influence of biogeography on microbial community composition and diversity, we could identify a core microbiome associated with *A*. *dealbata* (*sensu* ref. 44). In total, 1,900 bacterial OTUs (6% of total OTUs, 72% of total reads) and 109 fungal OTUs (0.7% of total OTUs, 43% of total reads) were identified in rhizosphere soils in all eight locations, suggesting that there is a set of *A*. *dealbata*-associated microbes across a wide array of environments. Of those core OTUs, a total of 141 bacterial taxa and 14 fungal taxa were identified as biomarkers of the rhizosphere (Fig. 5 and Supplementary Dataset S2). The most important bacterial biomarkers of the rhizosphere were *Proteobacteria* classified as *Bradyrhizobium* (*Alphaproteobacteria*, 2 OTUs) and *Burkholderia* (*Betaproteobacteria*, 2 OTUs). Several species of the genus *Burkholderia* are known for their plant growth-promoting activities^45^ and ability to form rhizobial symbiotic relationships with several South African legumes^46^. *Bradyrhizobium* species are the predominant N_2_-fixing rhizobia associated with acacias (e.g., refs 47 and 48). N_2_-fixing symbionts enhance growth and competitive ability of their host plants^49^, which consequently influences plant invasiveness^50^. Interestingly, it has been reported that seven of nine selected acacia species grew better in association with *Bradyrhizobium* phylotypes than with members of the genera *Burkholderia*, *Rhizobium* and *Ensifer* ^51^.

Two fungal OTUs belonging to the families *Pezizaceae* (*Pezizomycetes*, 1 OTU) and *Telephoraceae* (*Agaricomycetes*, 1 OTU), together with an OTU classified as *Inocybe sp*. (*Agaricomycetes*), were the most abundant in the rhizosphere of *A*. *dealbata*. Numerous *Pezizaceae* and *Telephoraceae* fungi and members of the genus *Inocybe* are defined as EMF^22^. EMF associate with a large number of woody plants, facilitating plant nutrient uptake, especially phosphorus^52^. In addition, EMF may indirectly contribute to the global N cycle by providing phosphorus and other nutrients to N_2_-fixing legumes, particularly in natural ecosystems that are often P limited^53^. Several fungal genera (*Clonostachys*, *Truncatella* and *Clonostachys*) catalogued as probable plant pathogens^54^ using FUNGuild were also found as biomarkers of the rhizosphere (Supplementary Dataset S2). Pathogens can potentially reduce the performance, and therefore impacts, of invasive species. Alternatively, pathogen accumulation may exacerbate the effects of invasions if pathogens negatively affect co-occurring native species and reduce their performance and competitive ability^55^.

Furthermore, functional analyses of shotgun sequencing data suggest significant enrichment of approximately 300 genes in rhizosphere compared to bulk soils (Supplementary Dataset S3), among them, genes associated with membrane transport, signal transduction, xenobiotic degradation and metabolisms, and genes encoding for the metabolism of carbohydrates, amino acids, iron and nitrogen. Similar findings have been reported in other studies^56, 57^, supporting the view that microbial communities in the rhizosphere are selected based on the functions they perform.

In summary, we have shown that *A*. *dealbata* associates with different bacterial and fungal communities, depending on biogeography and soil nutritional status. Nonetheless, a large group of taxa were shared across all locations; these taxa represented >50% (for bacteria) and >40% (for fungi) of the rhizosphere community. Some of these microbes are likely involved in nutrient cycling, whereas others may act as plant pathogens. This is relevant because it shows that different mutualists of the root microbiome of *A*. *dealbata* have the potential to complement each other in acquiring different limiting nutrients, which concomitant with the possible accumulation of plant pathogens of native plants may contribute to the widespread success of this invader in novel environments.

## Materials and Methods

### Study sites and sample acquisition

Soil was collected in October 2015 from eight sites (between 3 to 532 km apart) in the grassland biome of South Africa (Table 1 and Supplementary Fig. S1). In South Africa, *A*. *dealbata* mainly invades grassland riparian habitats^58, 59^. At each site two types of samples were collected: rhizosphere soil, which was firmly attached to the roots, and bulk soil, gathered 10–20 m away from any conspecific tree to avoid ongoing plant-soil microbial feedbacks^60^. Sampling and processing equipment was sterilized with 90% ethanol after each sampling regime. A 2-mm sieve was used to remove leaves and other coarse material prior to sample homogenization. Soil sub-samples for genetic analysis were stored at −80 °C, within three days of sampling, until further analysis. A total of 80 samples were collected (8 populations ×5 individuals ×2 habitat types (rhizosphere or bulk soil)).

**Table 1:** Geographic location, mean annual temperature (MAT), temperature seasonality (TS, standard deviation), minimum temperature of coldest month (MTCM), mean annual precipitation (MAP), precipitation seasonality (PS, coefficient of variation), precipitation of wettest quarter (PWQ), precipitation driest quarter (PDQ) and vegetation type of the study sites.

Province	Gauteng		KwaZulu-Natal			Mpumalanga		
Vegetation type^a^	Rand Highveld Grassland		Midlands Mistbelt Grassland			Eastern Highveld Grassland		
Location number	GP1	GP2	KN1	KN2	KN3	MP1	MP2	MP3
Latitude	−25.927	−25.914	−30.561	−30.101	−29.374	−26.1467	−25.940	−25.840
Longitude	28.488	28.514	29.814	30.025	30.023	29.768	29.945	29.241
MAT (°C)^b^	15.7	15.8	16.3	15.7	14.2	14.7	14.5	15.5
TS (SD)	3.9	3.9	2.9	2.9	3.7	3.7	3.6	4.1
MTCM	0.7	0.8	4	3	0	0.1	0.2	−0.4
MAP (mm)	706	704	913	871	865	733	759	694
PS (CV)	79	79	58	62	67	75	74	74
PWQ	363	361	385	379	407	374	385	344
PDQ	23	23	60	60	48	21	26	20

^a^Vegetation data was obtained from ref. 79.

^b^Climatic data was obtained from ref. 80.

### Soil chemistry analysis

Soil chemical analysis was performed according to standard methods at BemLab (SANAS Accredited Testing Laboratory, Cape Town, South Africa). pH was determined in saturated soil extracts (SSE). Total N and C were measured by combustion at 1350 °C. Total phosphorus, potassium, calcium and magnesium were extracted using HCl-HNO_3_ after combustion (3 h, 550 °C), followed by quantification by inductively coupled plasma optical emission spectrometry (ICP-OES). Ammonium and nitrate were extracted with 2 M KCl and diluted prior to determination by a flow injection analyser (FIA). Soil characteristics and all metadata are presented in Supplementary Dataset S1.

### DNA extraction and sequencing

Genomic DNA was isolated from the 80 samples using a PowerSoil DNA isolation kit (MO Bio Laboratories, Inc. Carlsbad, CA) following the manufacturer’s instructions. The internal transcribed spacer (ITS) region was amplified using fungal-specific primers^61^: ITS1F (5′-CTTGGTCATTTAGAGGAAGTAA-3′) and ITS4 (5′-TCCTCCGCTTATTGATATGC-3′). Bacterial 16S rRNA gene amplicons were amplified using primers 515F (5′-GTGYCAGCMGCCGCGGRA-3′) and 909R (5′-CCCCGYCAATTCMTTTRAG-3′) as in Oloo, *et al*.^62^. DNA regions were amplified using the HotStarTaq Plus Master Kit (Qiagen, Valencia, CA). Amplicons from different samples were mixed in equal concentrations and purified using Agencourt Ampure beads (Agencourt Bioscience Corporation, USA). Paired-end 2 × 250 bp sequencing was performed on an Illumina MiSeq instrument (Illumina Inc., San Diego, CA, USA).

Additionally, 4 bulk soil and 4 rhizosphere samples were randomly selected for shotgun metagenomic sequencing. Shotgun libraries were prepared using a Nextera DNA sample preparation kit (Illumina Inc., San Diego, CA, USA) following the manufacturer’s instructions. Libraries were sequenced using an Illumina Hiseq-2000 using paired-end technology (2 × 150 bases). All sequencing was performed by the Molecular Research LP next generation sequencing service (http://www.mrdnalab.com).

### Amplicon sequencing analysis

Sequence data were analysed using QIIME version 1.9.1^63^. Sequences that were <200 bp, contained more than 2 ambiguous characters, had quality scores <25, or contained more than one mismatch to the sample-specific barcode or to the primer sequences were excluded from further downstream analyses. Chimeric sequence detection and OTU selection at 97% sequence similarity were conducted using USEARCH v6.1^64^. Taxonomies were assigned to each OTU using the RDP Naïve Bayesian Classifier^65^ with the SILVA-ARB (release 123) and UNITE-INSD (release 7) databases for bacteria and fungi, respectively. OTUs whose classifications did not match their expected taxonomic kingdoms (fungi and bacteria, respectively) were removed. Singletons were excluded and each sample was randomly subsampled (rarefied) to the same number of sequences per sample; that is, 29,678 bacterial and 8,093 fungal sequences. Two samples with a low number of sequences were excluded, yielding a total of 78 (39 rhizosphere and 39 bulk soil) samples that were used for downstream analyses.

### Shotgun metagenomic analysis

Paired-end sequences were quality filtered with PRINSEQ^66^ and joined using FLASH^67^. Combined reads were aligned against the NCBI-NR protein database using DIAMOND BLASTX v0.7.1 (E-value cut-off at 1e-5)^68^. Functional annotation was performed based on KEGG pathways and SEED subsystems in MEGAN v5.0.3^69^. The matrix of raw counts of functional annotations were normalized to account for the unequal sequence coverage between samples.

### Statistical analyses

OTU richness and diversity indices (richness, Shannon, inverse Simpson and Pielou’s evenness), together with accumulation curves were calculated using the vegan R package^70, 71^. We applied a mixed model ANOVA to determine significant differences in microbial diversity and soil chemistry between rhizosphere and bulk soils using the phia package^72^. In these analyses, location was specified as a random factor. Abiotic data were standardized and pair-wise distances computed based on Euclidean distances. Community data matrices were Hellinger-transformed and the Bray-Curtis distance measure was used to generate a dissimilarity matrix. Weighted and unweighted UniFrac dissimilarities were also obtained^73^. The effect of abiotic data in explaining variation in microbial community structures was assessed by distance-based redundancy analysis. First, we checked for collinearity among variables. This led to the removal of Na (correlated with NO_3_; r^2^ = 0.64) and Ca (correlated with pH; r^2^ = 0.80). Then, we performed forward selection to select the best set of variables that could explain the variation in community composition. Variation partitioning analyses were performed to determine the respective effects of the environment (soil chemistry) and geographic distances on the variation in microbial community composition^74^. A permutational analysis of variance (PERMANOVA)^75^ was carried out to test for differences in composition between habitats (bulk and rhizosphere soil) using the ‘adonis’ function (strata = location) in vegan. Selection for putative major microbial players in the rhizosphere communities was carried out as follows. First, we identified rhizosphere bacterial and fungal taxa that were present in all eight locations. We then performed linear discriminant analysis (LDA) effect size (LEfSe)^76^ to discriminate between microbial markers of the rhizosphere and bulk soil. We hypothesised that if microorganisms are selected within a given habitat on the basis of their functional capacities, microbial markers should be among the most influential microbial taxa in their respective habitat. Finally, we plotted the average relative abundance and frequency of occurrence of those taxa across each sample type to infer their putative ecological relevance. FUNGuild^77^ was used to assign fungal phylotypes to one of three trophic modes (saprotroph, symbiont or pathogen) where possible. To determine statistical differences between the functional potential of the rhizosphere and the bulk soil samples, the Statistical Analysis of Metagenomic Profiles (STAMP^78^) software package was used. P-values were calculated using a two-sided Fischer’s exact test and corrected using Benjamini-Hochberg false discovery rate.

### Data availability

The raw sequencing reads for this project were submitted to the National Center for Biotechnology Information Short Read Archive under accession no. SRP090490 (targeting sequencing) and SRP098951 (shotgun sequencing).

## Electronic supplementary material

Supplementary Information

Dataset S1

Dataset S2

Dataset S3
