# Supplementary material for: Soil nutritional status and biogeography influence rhizosphere microbial communities associated with the invasive tree Acacia dealbata
Source: Sci Rep. 2017 Jul 26;7:6472. doi: 10.1038/s41598-017-07018-w (PMC5529528; doi:10.1038/s41598-017-07018-w)

Soil nutritional status and biogeography influence rhizosphere microbial communities associated with the invasive tree *Acacia dealbata*

Casper N. Kamutando, Surendra Vikram, Gilbert Kamgan-Nkuekam, Thulani P. Makhalanyane, Michelle Greve, Johannes J. Le Roux, David M. Richardson, Don Cowan, Angel Valverde

Supplementary Material (Figures)

Figure S1 Study sites and biogeography of *Acacia dealbata*. The region covered by the grassland biome in South Africa is showed in green. Location numbers are as in Table 1. The map was created in ArcMap v. 10.2 (http://desktop.arcgis.com/en/arcmap/) using data from Mucina, L. & Rutherford, M.C. (2006) The Vegetation of South Africa, Lesotho and Swaziland, South African National Biodiversity Institute, Pretoria, freely available at https://www.sanbi.org.

Figure S2 Venn diagram showing the number of shared total A) bacterial and B) fungal OTUs (both 97% cut-off). The percentage of sequences associated with OTUs is shown in parentheses.

Figure S3 Accumulation curve (mean ± 95% confidence interval) of bacterial and fungal OTU (both 97% cut-off) richness *vs.* number of samples.

Figure S4 Diversity measures (richness, Shannon, inverse Simpson and Pielou's evenness) of A) bacterial and B) fungal OTUs (both 97% cut-off).

Figure S5 Boxplot depicting differences in soil chemistry between habitats. Different letters above each box denote a significant mean difference between bulk (B) and rhizosphere (R) soils ($P < 0.05$).

Supplementary Figure S1

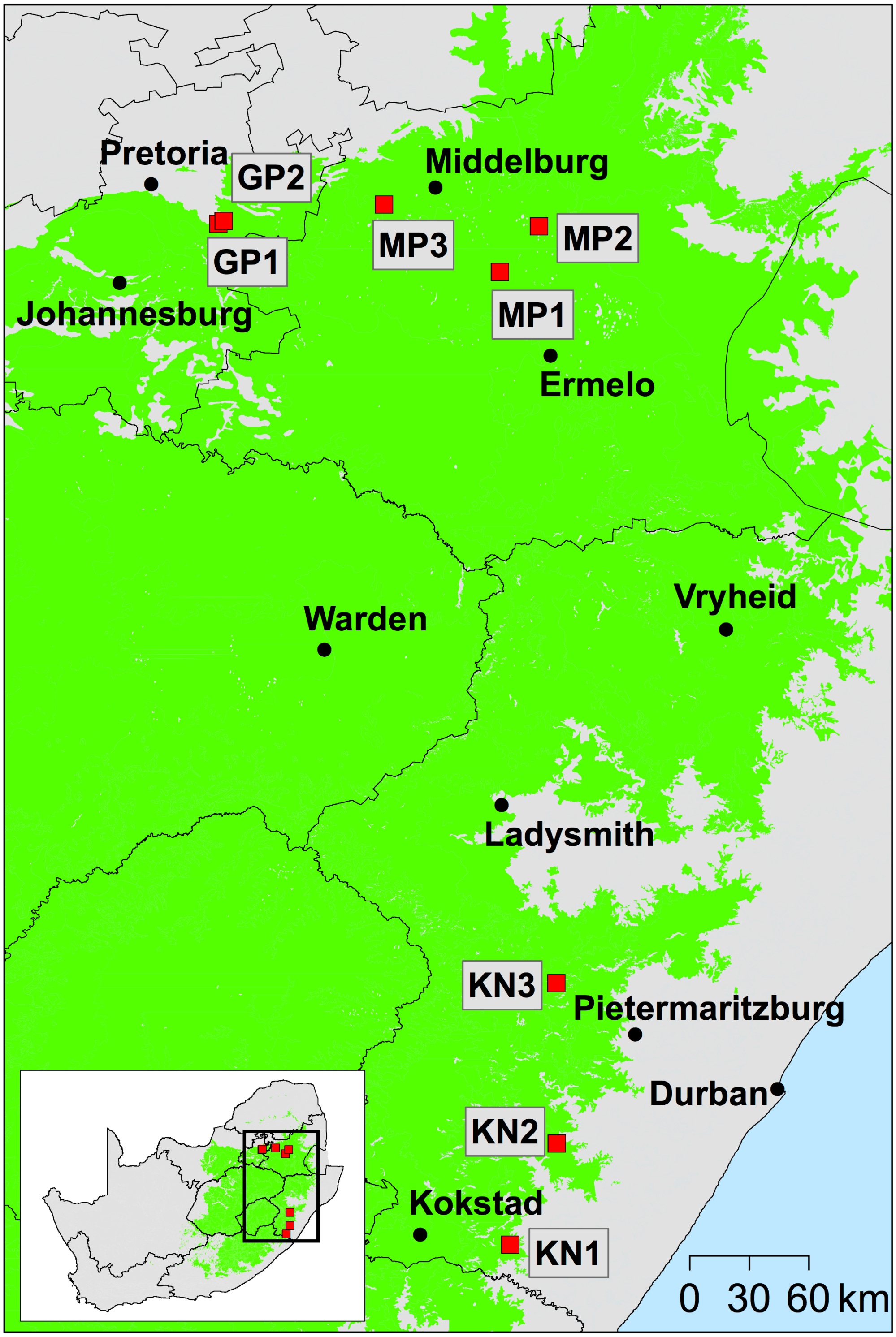

Supplementary Figure S2

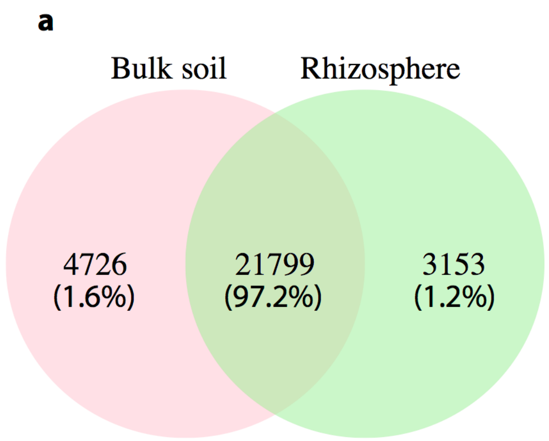

Supplementary Figure S3

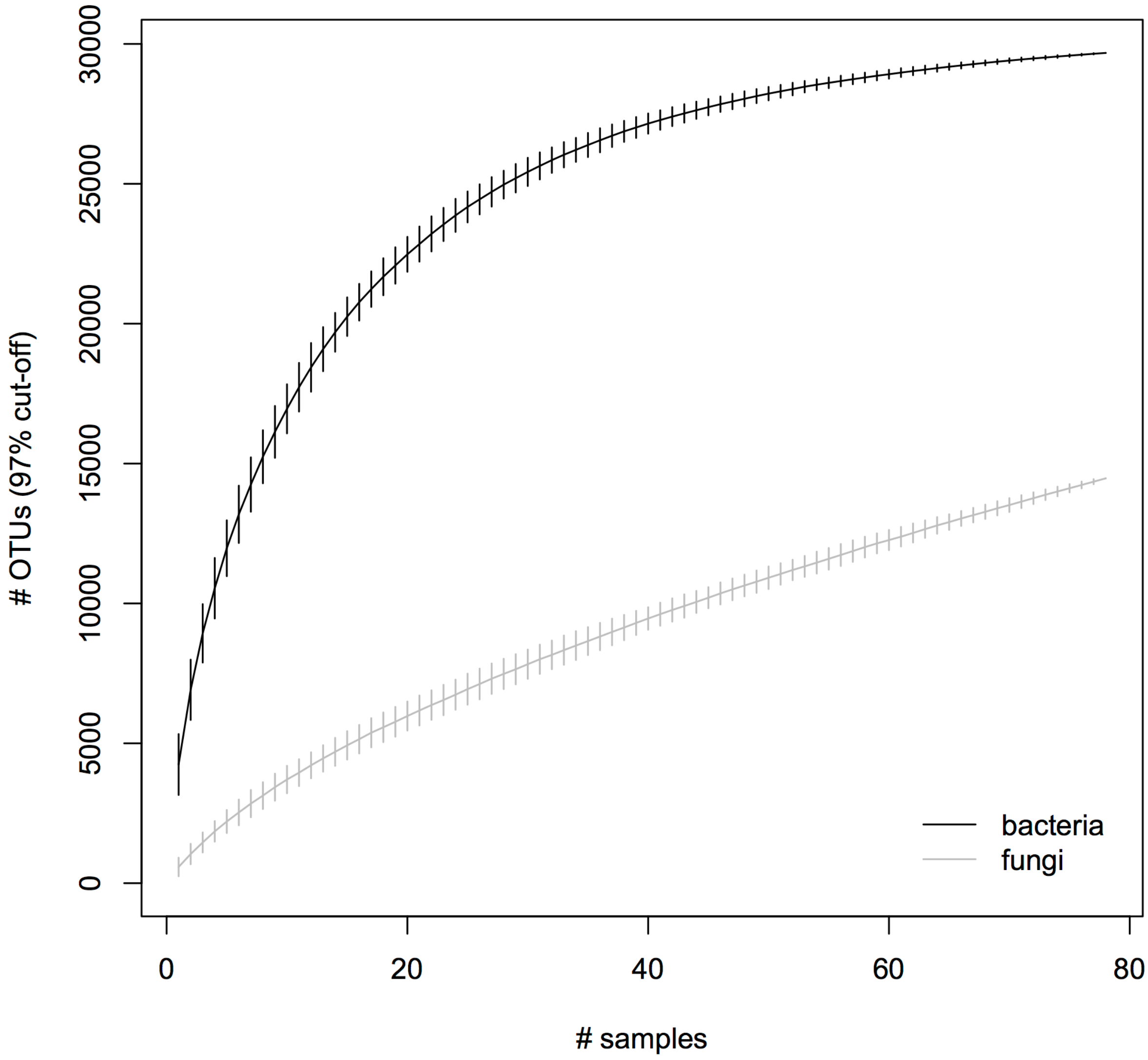

Supplementary Figure S4

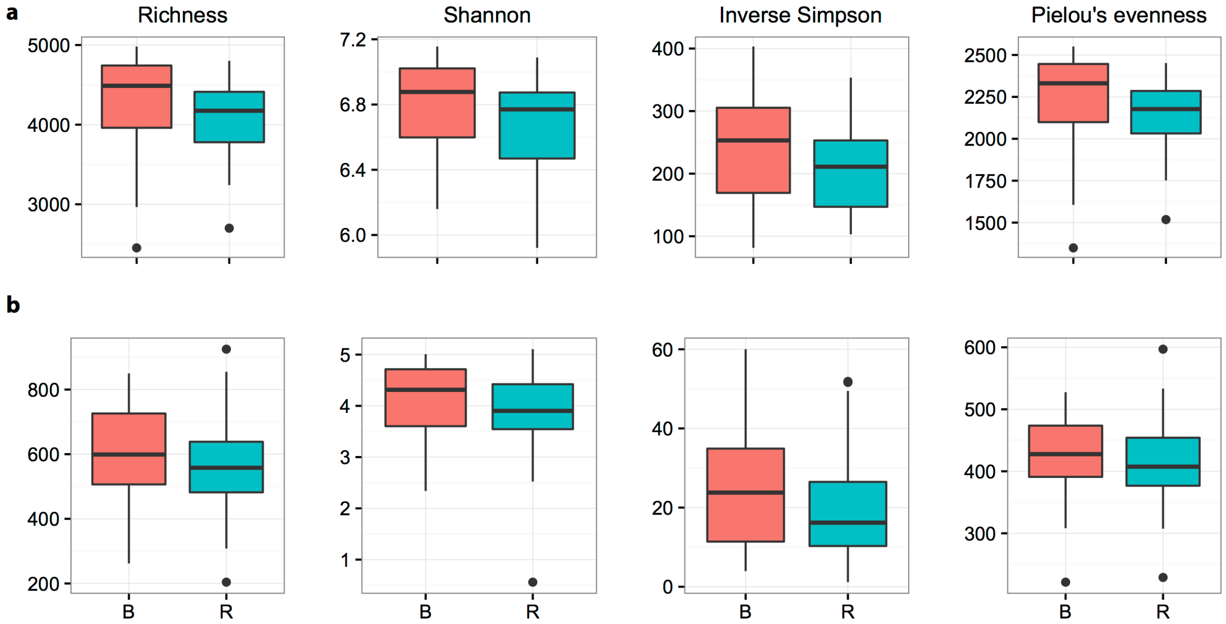

Supplementary Figure S5

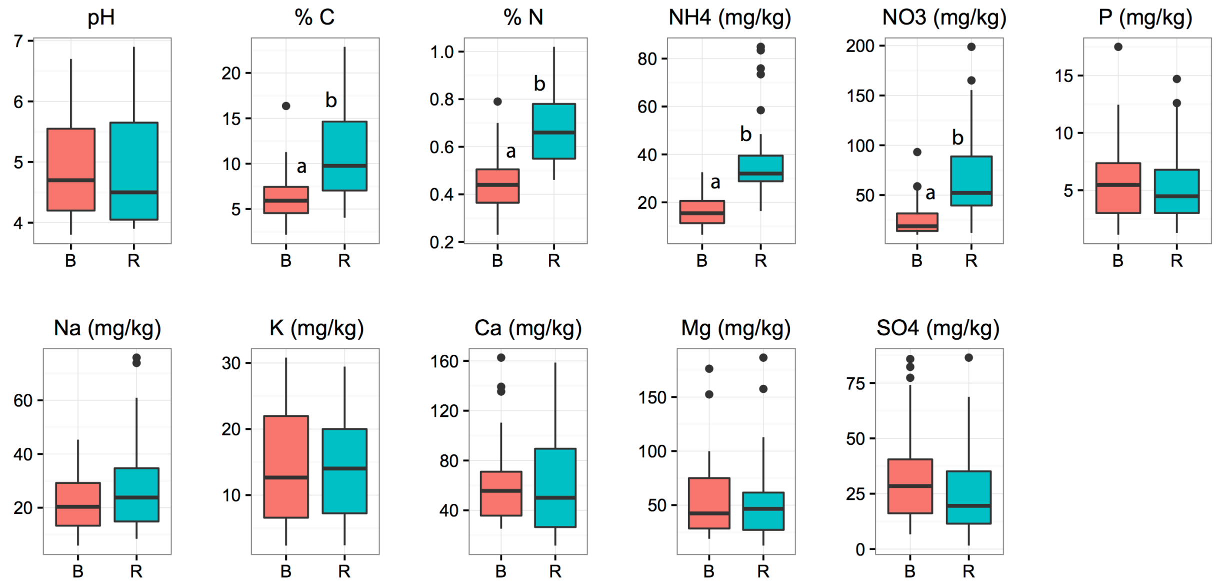

Supplement: Supplementary file 1 — Supplementary Information [file 41598_2017_7018_MOESM1_ESM.pdf]
